# MLN4924 (Pevonedistat), a protein neddylation inhibitor, suppresses proliferation and migration of human clear cell renal cell carcinoma

**DOI:** 10.1038/s41598-017-06098-y

**Published:** 2017-07-17

**Authors:** Shuai Tong, Yang Si, Hefen Yu, Lingqiang Zhang, Ping Xie, Wenguo Jiang

**Affiliations:** 1 Department of Biochemistry and Molecular Biology, Capital Medical University, Beijing, 100069 China; 2 Beijing Key Laboratory for Cancer Invasion and Metastasis Research, Capital Medical University, Beijing, 100069 China; 3 State Key Laboratory of Proteomics, Beijing Proteome Research Center, Beijing Institute of Radiation Medicine, Collaborative Innovation Center for Cancer Medicine, Beijing, 100850 China; 4 Cardiff China Medical Research Collaborative, Cardiff University School of Medicine, Heath Park, Cardiff, CF14 4XN UK

## Abstract

Neddylation is a post-translational protein modification associated with cancer development. MLN4924 is a neddylation inhibitor currently under investigation in multiple phase I studies on various malignancies, and its clincal name is Pevonedistat. It has been documented that MLN4924 blocks Cullins neddylation and inactivates CRLs and, in turn, triggers cell-cycle arrest, apoptosis, senescence and autophagy in many cancer cells. In this study, we investigated the anti-tumor effect of MLN4924 in human clear cell renal carcinoma (ccRCC). Levels of both Nedd8 activating enzyme E1 and Nedd8-conjugating enzyme E2 were higher in ccRCC tissues and RCC cancer cells than in normal. Moreover, MLN4924 treatment led to rapid inhibition of Cullin1 neddylation and notably suppressed growth and survival as well as migration in a dose-and time-dependent manner. Mechanistic studies revealed that MLN4924 induced the accumulation of a number of CRL substrates, including p21, p27 and Wee1 to trigger DNA damage and induce growth arrest at the G2/M phase. MLN4924 also induced anti-migration and anti-invasion by activating E-cadherin and repressing Vimentin. Taken together, this study provides the first evidence that neddylation pathway is overactive in ccRCC and that MLN4924 induces dose-dependent anti-proliferation, anti-migration, anti-invasion in ccRCC cells. The study thus indicates that MLN4924 has potential therapeutic value for the clinical treatment of renal cancer.

## Introduction

Kidney cancer is one of the most common human malignancies neoplasms, and more than 300,000 new patients are diagnosed worldwide each year^1^. In 2015, there were 62,000 estimated new cases and 14,000 deaths from cancers of kidney, of which >90% were clear cell renal cell carcinoma (ccRCC), which originates from the epithelial lining of the proximal convoluted tubules and is responsible for 60% to 80% of RCC among adults^2, 3^. Renal cell carcinomas are best treated by surgical resection, but approximately 30% of patients with metastatic renal cell carcinomas are not permissible to resection and have to mainly rely on traditional chemotherapies^3^. However, the commonly used chemotherapy for the treatment of metastatic carcinomas is far from satisfaction, especially for ccRCC patients. Traditional chemotherapy was mainly embodied with relatively low anticancer efficacy, acquired drug resistance, severe treatment-associated adverse effects, which leading to high risk of tumor recurrence and poor prognosis^4, 5^. The current dilemma makes it pressing issue in finding new anticancer targets and developing novel therapeutic agents with high efficient and less harmful side effects to improve the treatment of renal cancer.

Neddylation, adding Nedd8, an ubiquitin-like molecule, to target proteins, has been described as a post-translational protein modification back in 1997^6^. This reaction includes a three-step enzymatic cascade mediated by Nedd8-activating enzyme (composed of APP-BP1 and Uba3, E1), Nedd8-conjugating enzyme E2 (Ubc12 or Ube2F) and substrate-specific E3 ligases^7, 8^. Known physiological substrates of neddylation are Cullin family members. However, in recent years, more non-Cullin substrates have been identified. They include p53, MDM2, Smurf1, JunB and a few others^9–11^. Cullin neddylation leads to activation of Cullin-RING ligases (CRLs), the largest family of E3 ubiquitin ligases, which are responsible for ubiquitylation and degradation of many key signaling or regulatory proteins^8^. Through modulating CRLs, neddylation regulates several biological processes, including cell cycle, signal transduction, and tumorigenesis. It is anticipated that deregulation of CRLs is associated with uncontrolled proliferative diseases such as cancer. Among all CRLs, CRL1, also known as SCF (Skp1-Cullin1-F-box protein), is the best studied member of CRLs^12^. Dysfunction of CRLs, has been lined to human diseases, including cancer^13–15^.

MLN4924 is a specific small molecule inhibitor of NAE and has been advanced into several phase I clinical trials for certain solid tumors and hematologic malignancies because of its significant anticancer efficacy in preclinical studies^16^. The underlying mechanism of MLN4924 has been thought to be its inhibitory effects on NAE activities by binding to NAE to create a covalent Nedd8-MLN4924 adduct^17^. Consequently, MLN4924 efficiently blocks neddylation of all Cullins, leading to accumulation of their substrates^18–20^, which in turn triggers DNA replication stress, DNA damage response, cell-cycle arrest, apoptosis, autophagy, and senescence, collectively suppressing the growth of cancer cells^21–24^. Neddylation pathway components and CRL1/SCF E3 ligase are potential anti-cancer biomarkers, to which MLN4924 could serve as a promising drug for cancer therapy^25–30^. In renal cancer, a cancer type highly resistant to chemotherapy, the efficacy of MLN4924 is unknown but may be a significant interest. In this study, our data showed that MLN4924 markedly inhibited the growth of renal cancer cells by blocking Cullin1 neddylation and subsequent accumulation their substrates. This led to a DNA damage response, G2-M cell cycle phase arrest and apoptosis. What’s more, we found that MLN4924 blocked migration of renal cancer cells through upregulating E-cadherin and repressing of Vimentin. Collectively, our study demonstrated that MLN4924 effectively suppressed proliferation, survival and migration of renal cancer cells. The study thus provides proof-of-concept evidence for the clinical investigation of this first-in-class anticancer agent in the treatment of renal cancers.

## Results

### MLN4924 effectively inactivated Cullin1 neddylation in human renal cancer cells

**Figure 1:**
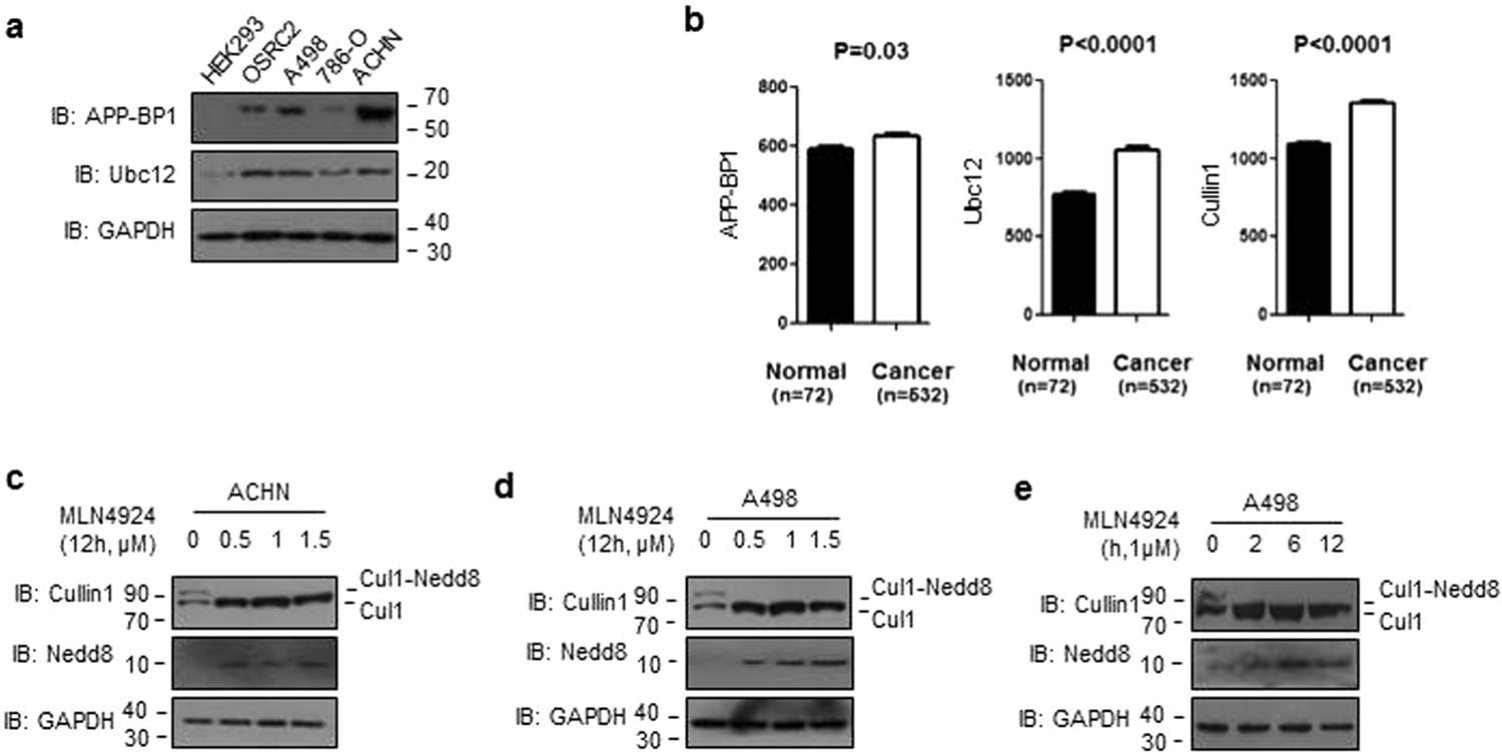
Expression of neddylation pathway proteins and MLN4924 effectively inhibited Cullin1 neddylation in human ccRCC cells. (**a**) Neddylation pathway proteins APP-BP1 (E1) and Ubc12 (E2) were analyzed by western blot using antibodies against indicated proteins. (**b**) The mRNA level of APP-BP1, Ubc12 and Cullin1 were analyzed in TCGA-KIRC dataset. Significance between the 2 populations was determined with a two tailed t-test. (**c,d**) Cells were treated with MLN4924 at indicated doses and for 12 hrs before being subjected to Western blot analysis. (**e**) Cells were treated with MLN4924 at 1 μM and for indicated dose-time before being subjected to Western blot analysis.

Previous studies have shown that Cullin1 was increased in renal cell carcinoma and associated with renal cancer cell proliferation, migration, and invasion^31^. However, there has no evidence to illustrate the relevance between neddylation pathway and ccRCC. To further investigate the neddylation pathway in ccRCC, we detected the protein level of APP-BP1 (neddylation E1), Ubc12 (neddylation E2) in HEK293, OSRC2, A498, 786-O and ACHN cells. Compared with the levels in immortalized “normal” human renal cell HEK293 cells, APP-BP1 and Ubc12 were high-expressed in most of renal cancer cell lines tested, with the highest expression seen in ACHN cells (Fig. 1a). We further examined mRNA levels of APP-BP1, Ubc12 and Cullin1 in TCGA_KIRC dataset. The mRNA levels of APP-BP1, Ubc12 and Cullin1 were higher in ccRCC (n = 532) than adjacent normal tissues (n = 72), revealing that neddylation pathway was overactivated in ccRCC (Fig. 1b). Given that there has been no previous studies on a potential correlation between Cullin1 neddylation and sensitivity to MLN4924 in renal cancer cells, we firstly tested four renal cancer lines, ACHN, OSRC2, 786-O and A498 for their response to MLN4924 in Cullin1 neddylation. As shown in Figs 1c,d and S1a,b, MLN4924 indeed caused a concentration dose-dependent inactivation of Cullin1 neddylation in all four renal cancer cells, as evidenced by reduced levels of neddylated Cullin1. We futher detected the total Nedd8 and found that the expression of Nedd8 was markedly enhanced in the MLN4924-treated cells compared to the controls (Fig.1c,d). Furthermore, MLN4924 also caused a time dose-dependent inactivation of neddylated Cullin1 signal and upregulation of Nedd8 (Fig. 1e). These results collectively indicates that inhibition of Cullin1 neddylation by MLN4924 is effective in human renal cancer cells.

### MLN4924 suppressed growth and survival of renal cancer cells

**Figure 2:**
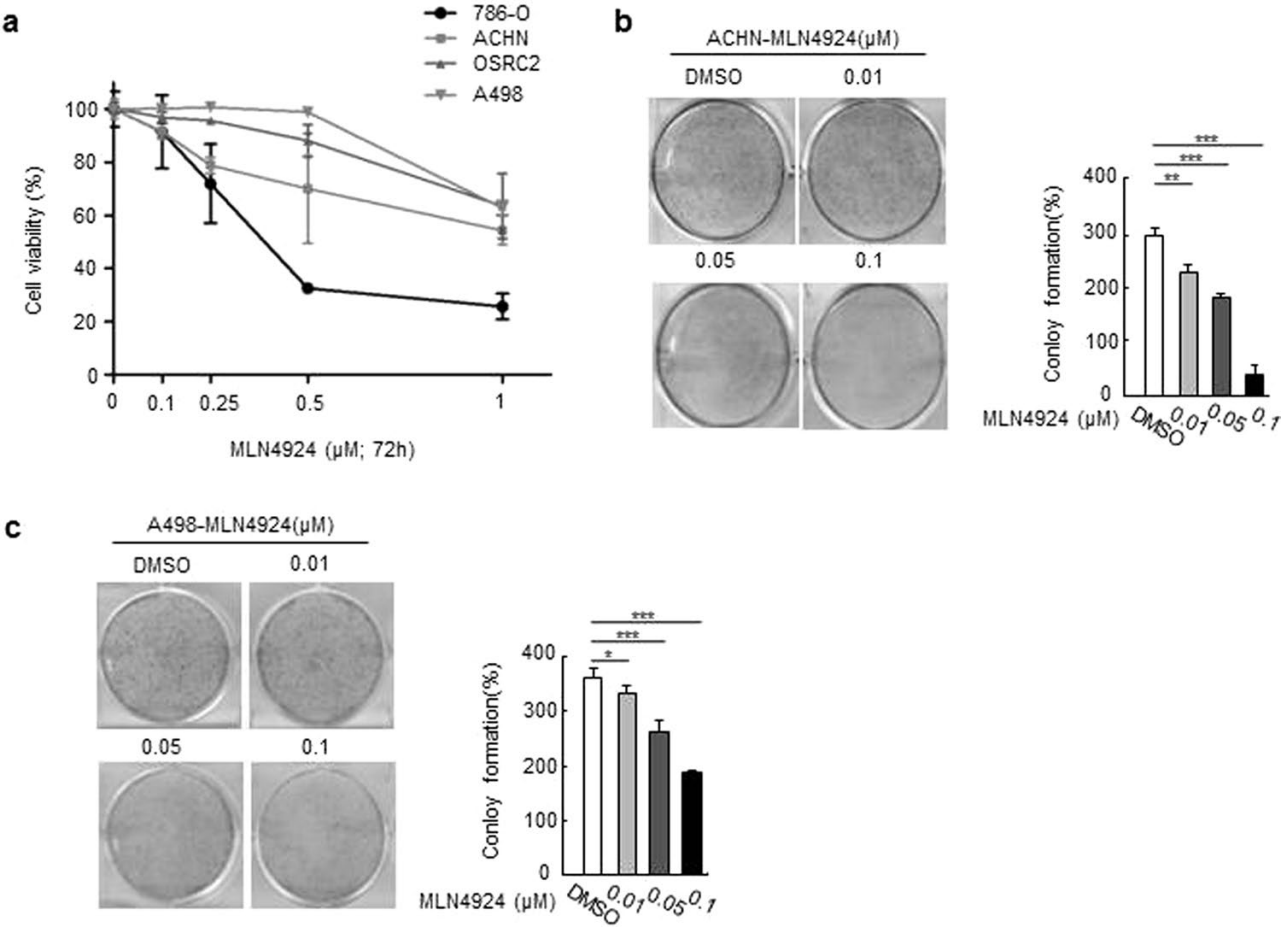
MLN4924 reduces human ccRCC cell viability. (**a**) Cells were treated with serial dilutions of MLN4924 for 72 hrs and cell viability was determined using CCK8 assays. Representative inhibitory curves from three independent experiments are shown for each cell lines. (**b**) Cells were seeded into 6-well plates petri-dishes at 500 cells per dish in triplicate and treated with MLN4924 for 12 days, followed by 0.01% (w/v) crystal violet staining and colony counting. Representative images of three independent experiments are shown for colony formation. (**c**) Graph of the relative number of colonies formed (the results of three independent experiments, expressed as mean ± SEM). (p < 0.05).

We next evaluated the ability of MLN4924 to reduce cell viability *in vitro* by treating the human renal cancer cell lines with serial dilutions of MLN4924 (0.1–1 μM) for 3 days. Indeed, 3 days of MLN4924 treatment showed a marked and dose-dependent reduction of cell viability in all four renal cancer cells (Fig. 2a). As a matter of fact, MLN4924 effect was very potent, but the drug sensitivity seems different in the four renal cancer cells. Notably, 1 μM MLN4924 almost completely inhibited cell viability in ACHN and 786-O cells, the effect on A498 and OSRC2 were comparably weak. Considering that ACHN cell line was derived from metastatic renal adenocarcinoma, it indicated that MLN4924 may intend to be more efficient and sensltive to treat invasive renal cancer.We chose to perform subsequent experiments on ACHN and A498 cells, as the former are more susceptible to MLN4924 treatment, while the latter are more resistant. We further carried out clonogenic assays to determine the long-term anti-proliferative effects of MLN4924 in ACHN and A498 cells. Twelve days of MLN4924 treatment strongly inhibited clone formation in a dose-dependent manner in both cell lines (Fig. 2b,c). Specifically, 0.01 μM MLN4924 reduced clone numbers, and 0.1 μM MLN4924 completely blocked clone formation. Taken together, these findings demonstrate that inhibiting the neddylation pathway with MLN4924 effectively reduces survival and growth of renal cancer cells.

### MLN4924 inhibited the degradation of CRL substrates and induces G2/M arrest in renal cancer cells

**Figure 3:**
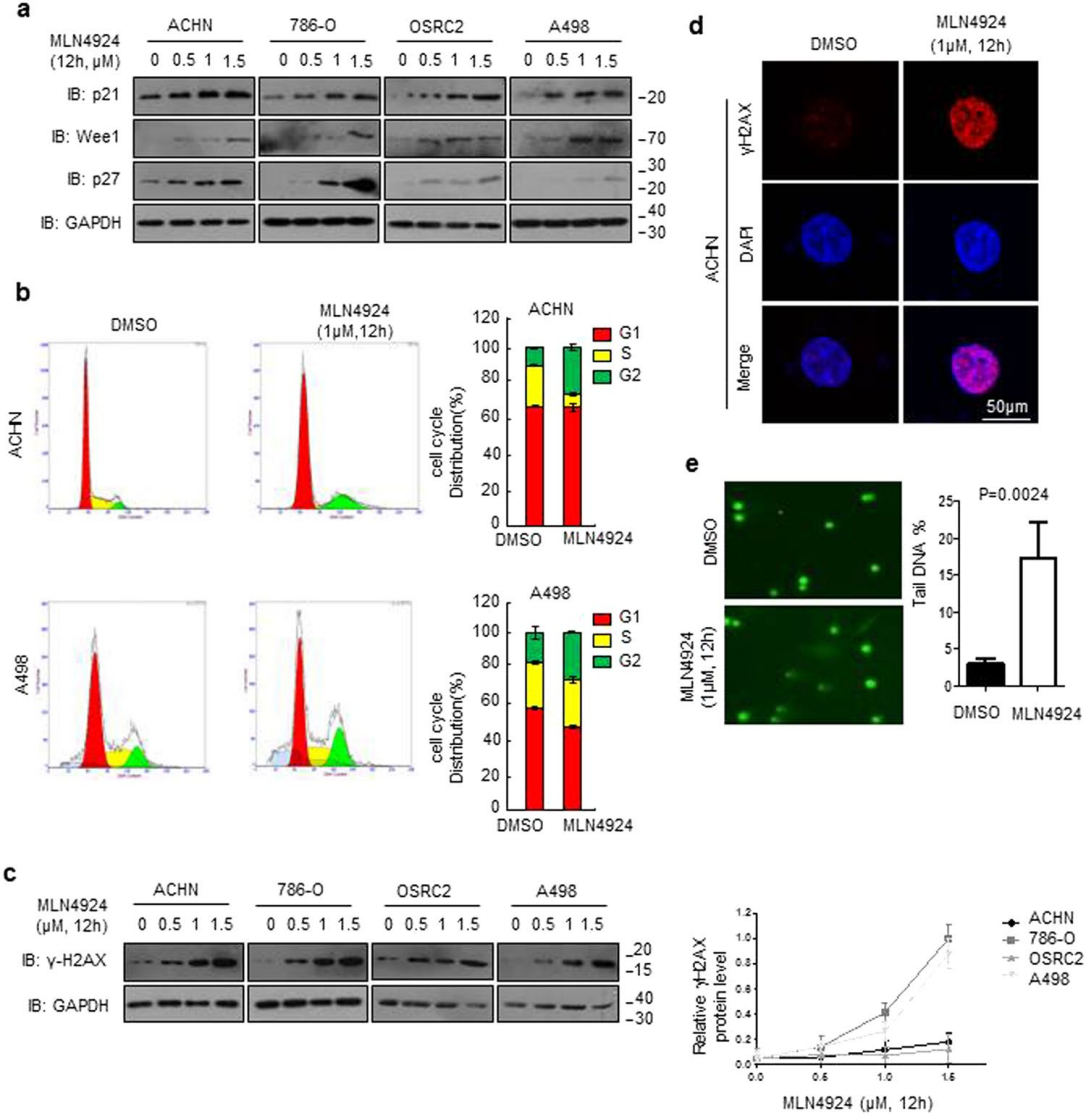
MLN4924 induces accumulation of CRL substrates in ccRCC cells. (**a,c**) Cells were treated with increasing concentrations of MLN4924 as indicated. Levels of p21, p27, Wee1 and γ-H2AX were examined by western blotting analysis in the whole cell lysates. (**b**) MLN4924 arrests cell cycle in the G2 phase in ccRCC cells. Cells were treated with DMSO control or MLN4924 at indicated concentrations for 12 hrs before subjected to flow cytometry assays. (**d**) MLN4924 led to DNA damage in ccRCC cells. ACHN cells were treated with MLN4924 at 1 μM and for 12 hrs, γ-H2AX staining was detected by immunofluorescence. (**e**) ACHN cells were treated with 1 μM MLN4924 for 12 hrs. The DNA tail moment for each experimental condition was quantified by alkaline comet assay. Representative images are shown. (the results of three independent experiments, expressed as mean ± SEM). (p < 0.05).

To determine the mechanisms underlying MLN4924-induced inhibition, we treated renal cancer cells with three sub-toxic concentrations of MLN4924 (0, 0.5, 1, 1.5 μM) for 12 hrs and detected the effects on levels of tumor-suppressive CRLs substrates using western blotting analysis. When the CRL E3 ligase function was inhibited by MLN4924, the degradation of CRLs substrates would also be inhibited. Indeed, MLN4924 treatment led to rapid accumulation of a panel of CRLs substrates, including p27, p21 and Wee1 (Fig. 3a), all being known tumor suppressive proteins^21, 26, 32^. It is noteworthy that MLN4924 increased p27, p21 and Wee1 levels very sharply and rapidly, even at a low concentration. MLN4924 decreased the turnover of p27, p21 and Wee1, thereby leading to an accumulation of their protein levels. The same effects of MLN4924 on CRLs substrates were observed in all the four kind of renal cancer cells. These results indicate that inhibition of Cullin neddylation by MLN4924 causes accumulation of tumor suppressive proteins in renal cancer cells. Since MLN4924 increased the levels of several critical cell cycle regulators, including p21, Wee1 and p27, we next examined whether these alterations resulted in cell cycle arrest in renal caner cells. MLN4924 increased the population of cells in the G2 phase while reducing the S phase population in ACHN cell line, which seems to be more sensitive to the treatment of MLN4924. However, the change of cell cycle distribution is very minor in A498 cell line (Fig. 3b).

### MLN4924 induced DNA damage in renal cancer cells

To evaluate whether MLN4924 might cause DNA damage in renal cancer cells, we examined the levels of γ-H2AX (phosphorylated H2AX (S139)), a typical bio-marker of DNA damage^33^. After MLN4924 treatment, there was an marked increase in γ-H2AX levels in renal cancer cell lines, compared with DMSO control. We thus perform statistical analysis to validate the levels of γ-H2AX and MLN4924 treatment upregulated the expression of γ-H2AX (Fig. 3c). Immunofluorescence staining further confirmed that MLN4924 substantially increased γ-H2AX fluorescence in the ACHN cell lines as compared with DMSO control (Fig. 3d). To confirm the cooperative DNA damaging properties of MLN4924, we conducted alkaline comet assays to quantify the impact of drug treatment on the tail moment in ACHN cells. Our results were consistent with those of our γ-H2AX immunocyto-chemistry analyses in that exposure to MLN4924 induced higher levels of DNA damage than untreated control (Fig. 3e). These data collectively showed that MLN4924 triggered DNA damage in renal cancer cells, and we speculated that MLN4924-induced DNA damage might be a common response to the upregulation of Wee1, p21 and p27.

### MLN4924 triggers apoptosis in renal cancer cells

**Figure 4:**
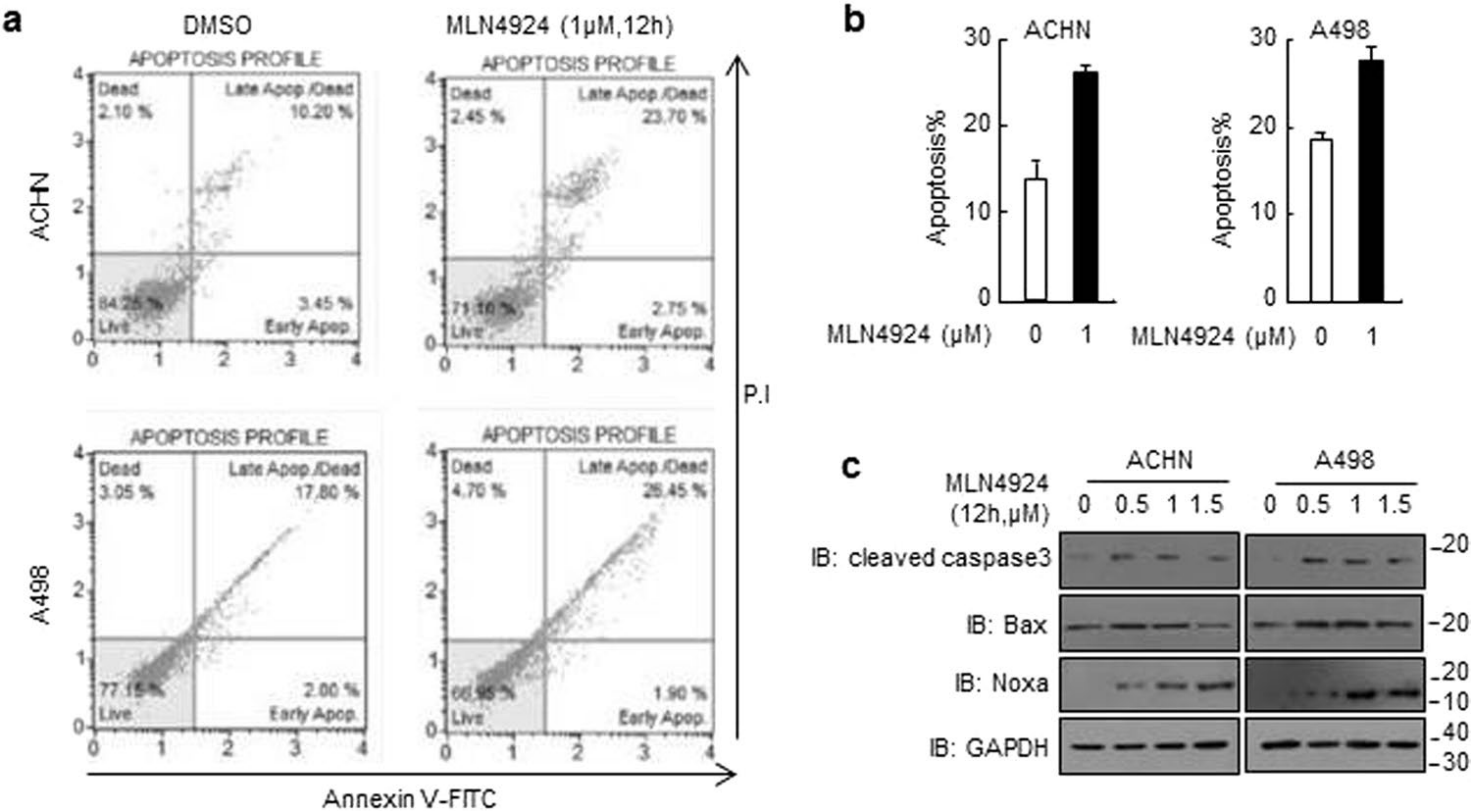
MLN4924 induces cell apoptosis in ccRCC cells. (**a**) Cells were treated with MLN4924 for 12 hrs, stained with Annexin-V-FITC and PI, and examined with flow cytometry assays. The percentages of ACHN and A498 cells in apoptosis are shown in (**b**). (**c**) Cells were treated with MLN4924 for 12 hrs, and levels of actived-caspase-3, Bax and Noxa were analyzed by western blotting in the whole cell lysates.

Because MLN4924 induced notable occurrence of DNA damage, we next examined the apoptotic effect of MLN4924 in renal cancer cell lines by using Annexin V-FITC/PI labeling flow cytometry. As shown in Fig. 4a and b, treatment with MLN4924 at 1 μM for 12 h induced apoptosis in 26.45% of ACHN cells, and in 28.35% of A498 cells. The same effect was also detected in OSRC2 cells (Fig. S2a). Under these conditions, the levels of apoptosis-associated proteins, caspase-3, Noxa and Bax proteins, were also seen to increase by MLN4924 (Fig. 4c).

### MLN4924 suppressed migration by up-regulating E-cadherin and down-regulating Vimentin

**Figure 5:**
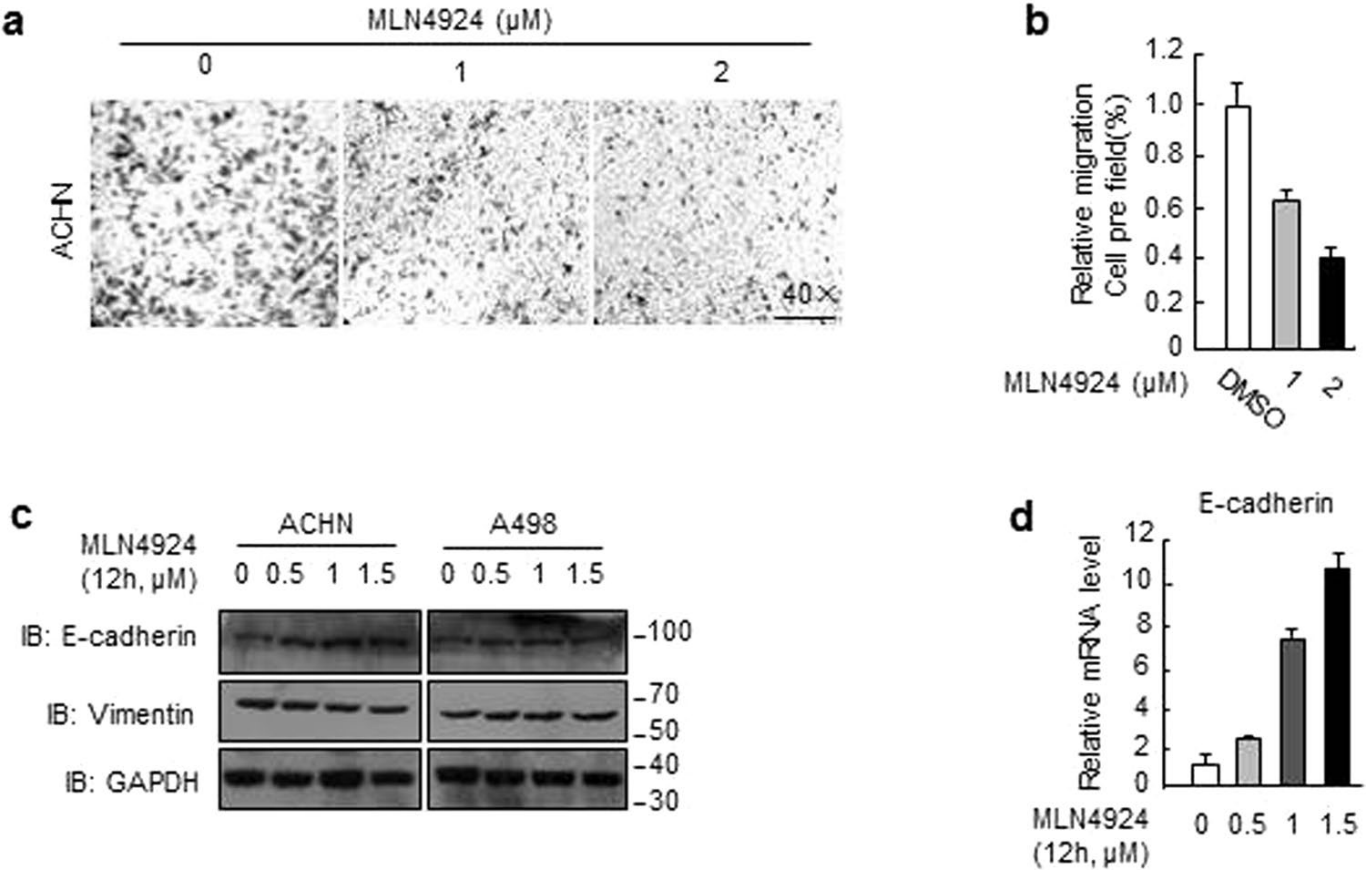
MLN4924 suppressed migration by up-regulating E-cadherin and down-regulating Vimentin. (**a**) Cells were treated with indicated concentrations of MLN4924 for 12 hrs before being subjected to Transwell migration analysis. Shown are representative images (**b**) or mean ± SD from 200 cells per well in triplicates. Similar results were obtained in three independent experiments. (**c**) Cells were treated with MLN4924 at indicated concentrations and subjected to Western blot analysis. (**d**) Cells were treated with MLN4924 at indicated concentrations, followed by total RNA isolation and qRT-PCR analysis for E-cadherin. Shown is mean ± SD. Similar results were obtained in three independent experiments.

To explore whether MLN4924 affects migration of renal cancer cells, we used the Transwell migration assays as functional readouts. ACHN has been observed to possess more significant cell migration and invasion, and we therefore determined the effects of MLN4924 on cell migration with this cell line by Transwell migration assay. Cells were starved for 24 hours prior to the experiment, and then various concentrates of MLN4924 were added to the upper chamber of Transwell migration chambers, and the concentration and duration used for MLN4924 treatment is non-toxic to cells. The lower chamber was filled with 600 μl medium containing 20% fetal bovine serum. After 18 hrs incubation, the cells that infiltrated the filter were counted in four random fields under microscopy. Indeed, MLN4924 significantly reduced cell migration in a dosage-dependent manner in ACHN cells (Fig. 5a,b). To elucidate underlying mechanism, we focused on potential changes in the levels of biomarkers for epithelial-to-mesenchymal transition (EMT). MLN4924 increased the expression of E-cadherin, an epithelial biomarker in a dosage-dependent manner in both renal cancer cell lines. Meanwhile, we also detected a down-regulating of Vimentin, a mesenchymal marker undergoing an EMT during the metastatic progression (Fig. 5c). To determine whether altered degradation of E-cadherin could be responsible for its accumulation upon MLN4924, we measured protein half-life of E-cadherin by blocking new protein synthesis using cycloheximide (CHX) and found that MLN4924 had minimal effect on E-cadherin degradation (Fig. S3a). We next determined potential effect in E-cadherin transcription and found that MLN4924 dramatically increased E-cadherin mRNA level in a dosage-dependent manner (Fig. 5d), suggesting the transcriptional activation is accountable for its accumulation upon MLN4924 treatment. Taken together, our results show that MLN4924-induced suppression of cell migration is likely mediated by transcriptional upregulation of E-cadherin.

## Discussion

The past decade has witnessed a rapid development in identifying new targets and hence new targeted therapies for for renal cancer. These drugs can be effective against advanced cancer, but unable to stop the tumour returning. Another set of drugs, which turns the body’s immune system against the tumours, is also offering hope, but has not been widely used. All these have rendered surgical resection being the main treatment options for the patients. However, at the time of diagnosis approximately 25–30% patient already present metastasis, reducing the overall survival of these patients to below 2 years^3, 34^. In addition, 20–50% of patients with localized disease eventually develop metastasis after nephrectomy with poor survival rate^35^. Disappointedly, there are no chemotherapies for this condition For this reason, renal cancer patients are more urgently in calling for novel therapeutic agents with high efficacy and low toxicity. and targeting Cullin RING ligases and neddylation, over-activated in various human cancers, could be a potential consideration. Recent studies have shown targeting CRL via inhibiting neddylation pathway by a small molecule inhibitor MLN4924 is an effective anticancer approach, as demonstrated in both preclinical and clinical settings^16, 36–39^.

In this study, we focused on whether MLN4924 could be therapeutically beneficial for human renal cancer. Here and for the first time, we have demonstrated that the neddylation pathway is activated in most renal cancer cells, as evidenced by comparative analysis of APP-BP1 and Ubc12 levels in renal tumor tissues and in healthy bone tissues. The increased mRNA levels of Ubc12, Cullin1 and APP-BP1 have been observed in TCGA-KIRC database, revealing that neddylation pathway is overactivated in ccRCC. Together, these findings indicate that neddylation plays a key role in sustaining ccRCC survival and suggest that MLN4924 might help treat ccRCC in most patients.

We have further demonstrated that MLN4924 prominently reduces the neddylation modification of Cullin1 and the proliferation of several renal cancer cells. This is likely due to its induction of cell cycle arrest, DNA damage and apoptosis in the cells. We argue that this is due to the accumulation of Cullin1 downstream substrates and critical cell cycle regulators, p21, p27, and G2 cell cycle checkpoint Wee1 by the compound.

There have been several lines of evidence from our study supportting the essential role of apoptosis in MLN4924-mediated anti-tumor activity. This study has shown that MLN4924 treatment activates apoptosis signaling, including the cleavage of caspase3, Bax and pro-apoptotic BH3-only protein Noxa induced apoptotic signaling^40^. Previous studies have showed, in several other cancers, that the accumulation of the Noxa is the mainly mechanism that MLN4924-induced apoptosis^41, 42^. Hence, we speculate that Bcl-2 protein member Bax is also important for MLN4924-induced apoptosis. Therefore, our data support the conclusions of previous studies in other cancers that MLN4924 inhibits cell proliferation via multiple pathways^25–29, 36–39^.

Our experimental data have also clearly demonstrated that renal cancer cell lines vary in their sensitivity to MLN4924 treatment. Amongst these cell lines, ACHN is the most sensitive to MLN4924. As shown in Figs 1c and 2b, a low concentration treatment of MLN4924 totally abolishes the activity of Cullin1 in ACHN cells, and dramatically inhibits the cell proliferation. Since the ACHN cell line was derived from metastatic renal adenocarcinoma and is highly aggressive in nature, we speculate that MLN4924 is more efficient and in treating invasive and metastasized renal cancer.

Our study has further disclosed that MLN4924 suppressed migration of ccRCC via multiple mechanisms, by regulating key player protein E-cadherin at transcriptional levels. The same has been reported in gastric cancer cells^26^. E-cadherin is the central mediator of cellular adhesion junctions and is required for the maintenance of the epithelial phenotype. In contrast, Vimentin, a marker of mesenchymally-derived cells or cells undergoing an epithelial-to-mesenchymal transition (EMT) during both normal development and metastatic progression^43^, can be down-regulated by MLN4924 treatment. Thus, the present study has discovered a previously unknown function of neddylation, namely the regulation of epithelial-to-mesenchymal transition (EMT), an interesting subject for future investigation.

In summary, our study proposes the following working model: Through inhibiting neddylation E1, MLN4924 inactivates CRL by blocking Cullin neddylation, which is followed by accumulation of many CRL substrates in a cell type and context dependent manner. Accumulation of p21, p27, Wee1 would cause DNA damage and trigger DSB response leading to G2 arrest. The sustainable treatment of MLN4924 led to apoptosis of tumor cells with the upregulation of Noxa and Bax. Finally, MLN4924 activates the expression of E-cadherin and inhibits Vimentin via a yet-to-be identified mechanism to remain cancer cells in an epithelial phenotype to prevent EMT. The final outcome of these comprehensive MLN4924 effect in ccRCC is effective suppression of proliferation, survival and migration. Overall, our study provides the proof-of-concept evidence for future development of neddylation inhibitors as a novel class of targeted therapy for the treatment of ccRCC patients.

## Methods

### Cell lines and chemicals

The ccRCC cell lines ACHN, OSRC2, 786-O, and A498 were obtained from American Type of Cell Collection (ATCC, Manassas, VA, USA) or China Infrastruture of Cell line Resources (Beijing, China) and maintained in PRMI1640 (HyClone, Beijing, China) supplemented with 10% fetal bovine serum (HyClone, Beijing, China). MLN4924 was purchased from Selleck Shanghai (Shanghai, China), and was dissolved in dimethyl sulfoxide (DMSO) and stored at −20 °C. Cycloheximide was purchased from Sigma (R750107).

### Antibodies and reagents

Anti-Cullin1, E-cadherin, Vimentin antibodies were purchased from Abcam (Cambridge, MA, USA). Antibodies against p21, p27, Wee1, Noxa, Caspase3, Bax, γ-H2AX were obtained from Cell Signaling Technology (Danvers, MA, USA). Anti-glyceraldehyde 3-phosphate dehydrogenase and secondary antibodies were produced by Santa Cruz Biotechnology (Santa Cruz, CA, USA).

### The Cancer Genome Atlas (TCGA)

The mRNA data (RNA Seq v2) for patients in the Cancer Genome Atlas_kidney renal clear cell carcinoma (TCGA-KIRC) dataset were downloaded from https://www.synapse.org/ and used for differential mRNA expression analysis.

### Cell viability assay

Cell viability was measured using a tetrazolium salt (WST-8)-based colorimetric assay in the CCK-8. Briefly, cells were seeded on 96-well plates at an initial density of 1 × 10^4^ cells per well. At the time points indicated, spent medium was replaced with fresh medium containing 10 ml CCK-8 solution, and the plate was incubated for 50 min. Cell viability was detected by scanning with a microplate reader at 450 nm.

### Clonogenic survival assay

500 cells were seeded into 6-well plates petri-dishes treated as indicated, and maintained for 12 days. Cells were then stained with 0.01% (w/v) crystal violet, and cell colonies were counted. The assays were performed in duplicate with at least three replications per treatment.

### FACS (fluorescence-activated cell sorting) analysis

Cells were treated with MLN4924 for 12 h, harvested and fixed in 70% ethanol overnight. Cells were washed twice with ice-cold PBS and then stained with propidiumiodide (PI, 20 mg/ml, Sigma) solution for 30 min in the dark. The samples were then analyzed using a Muse® Cell Analyzer (Merck) for cell cycle distributions. Apoptosis were detected by translocation of phosphatidylserine to the cell surface using an Annexin V-FITC apoptosis detection kit I (Muse®Annexin V & Dead Cell Assay Kit. Cat. No. MCH100105). Cells were collected and stained with FITC Annexin V and PI according to manufacturer’s instructions prior to analysis by Muse® Cell Analyzer.

### Cell migration and invasion

Boyden Chambers (24-well, 8 mm; Corning, Corning, NY, USA) were used to measure cancer cell migration and invasion. Cells (5 × 10^4^) in 100 μl serumfree RPMI 1640 medium were placed in the upper chamber, and 500 μl RPMI 1640 containing 10% fetal bovine serum was added to the lower compartment as a chemoattractant. After incubation for 36 hrs at 37 °C, cells on the top surface of the membrane were removed by wiping with a cotton swab. The migrated cells on the bottom surface of the membrane were fixed and stained with crystal violet. Cells were counted in four randomly selected fields. The invasion assay was performed in chambers coated with matrigel basement membrane matrix.

### Confocal microscopy, immuno-fluorescence analysis

For immuno-staining of endogenous γ-H2AX, ACHN cells were fixed in 4% PFA (paraformaldehyde) for 10 min, then fixed in 0.1% PBST (containing 0.5% Triton X-100) for 15 min. Further processing included incubating cells in 5% BSA for 10 min before incubations with γ-H2AX antibody for 3 hrs at 37 °C and with secondary antibody (Alexa Fluor® 594 Conjugate, Cell Signaling Technology) for 1 h at room temperature. Cells were analysed in PBS when the nucleus was stained with DAPI. Images of fixed cells were acquired on a confocal microscope using Laser Sharp software.

### RNA extraction and qRT–PCR

Total RNA was isolated with TRIzol reagent using to the manufacturer’s instructions. Quantitative real-time PCR was carried out as described previously^10^. The primers were listed in supplemental table1.

### Alkaline comet assays

Put the microscope slides into 37 °C overnight after immersed in the boiling normal melting point agarose (the concentration was 0.2%, diluted with PBS) for 1 minute, covered the microscope cover glasses on slides and chilled at 4 °C for 15 minutes after dropped normal melting point agarose (the concentration was 0.5%, diluted with PBS) on slides, covered glasses for 15 minutes at 4 °C. Test samples (ACHN cells were treated with 1μΜ DMSO or MLN4924 for 12 hrs) were respectively mixed with molten low melting point (LM) agarose (the concentration was 1%, diluted with PBS) and spread onto slides, then covered the glasses again gently for 15 minutes at 4 °C. Slides were incubated in lysis solution (2.5 mM NaCl after uncovered the glasses for 1 h at 4 °C, and then immersed in the cold electrophoretic buffer (300 mM NaOH, 1 mM EDTA) for 12 minutes. Further, slides were electrophoresed for 20 minutes at 300 mA. Following electrophoresis, microscope slides were immersed twice in neutralization buffer for 15 minutes. Next, microscope slides were incubated with 25 μl/ml Hoechst dyestuff for 10 minutes at 37 °C in the dark. Cells were imaged using fluorescent microscopy and tail moments from 50 cells per slide were calculated.

### Statistical analysis

Statistical analyses were performed using the SPSS 19.0 (SPSS Inc, Chicago, IL) and Graphpad Prism 5 (Graphpad software Inc, San Diego, CA). Group distributions were compared using the Student’s *t* test. A value of *P* <0.05 was considered statistically significant.

## Electronic supplementary material

Supplementary information
