# Supplementary material for: MLN4924 (Pevonedistat), a protein neddylation inhibitor, suppresses proliferation and migration of human clear cell renal cell carcinoma
Source: Sci Rep. 2017 Jul 17;7:5599. doi: 10.1038/s41598-017-06098-y (PMC5514088; doi:10.1038/s41598-017-06098-y)

Supplementary Information

MLN4924 (Pevonedistat), a protein neddylation inhibitor, suppresses proliferation and migration of human clear cell renal cell carcinoma

Shuai Tong[1,2], Yang Si[1,2], Hefen Yu[1,2], Lingqiang Zhang[3], Ping Xie[1,2*], Wenguo Jiang[1,2,4*]

[1]Department of Biochemistry and Molecular Biology, Capital Medical University, Beijing 100069, China.

[2]Beijing Key Laboratory of Cancer & Metastasis Research, Capital Medical University, Beijing 100069, China.

[3]State Key Laboratory of Proteomics, Beijing Proteome Research Center, Beijing Institute of Radiation Medicine, Collaborative Innovation Center for Cancer Medicine, Beijing 100850, China.

[4]Cardiff China Medical Research Collaborative, Cardiff University School of Medicine, Heath Park, Cardiff CF14 4XN, UK.

Correspondence to: *xpxp922@163.com; jiangw@cf.ac.uk.

S1

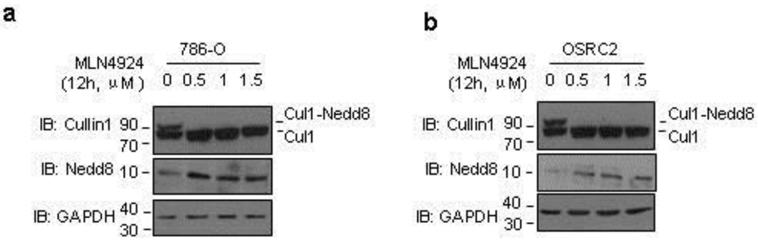

Figure S1. MLN4924 effectively inhibited Cullin1 neddylation in human ccRCC cells: Cells were treated with MLN4924 at indicated doses and for 12 hrs before being subjected to Western blot analysis.

S2

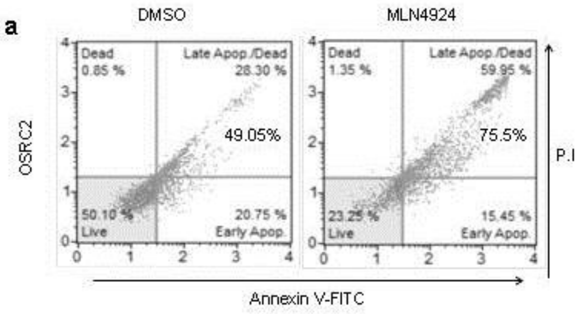

Figure S2. MLN4924 arrested cell cycle and induced apoptosis in ccRCC cells: (a) Cells were treated with DMSO control or MLN4924 at indicated concentrations for 12 hrs, stained with Annexin-V-FITC and PI, and examined with flow cytometry assays.

S3

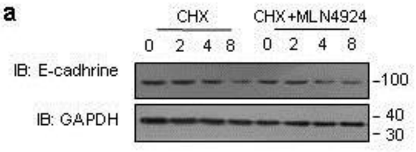

Figure S3. Effect of MLN4924 on protein half-life of E-cadherin and vimentin: Cells were treated with DMSO or MLN4924 containing cycloheximide (CHX, 10μM) for indicated time periods and harvested for Western blot analysis using indicated Abs.

Supplement Table 1. Primer sequences for qRT-PCR

Gene name	F or R Primer sequence
GAPDH	F: GGAGTCAACGGATTTGGT<br>R: GTGATGGGATTTCCATTGAT
E-cadherin	F: CAGAGCCTCTGGATAGAGAACGCA<br>R: GGCATTGTAGGTGTTCACATCAT

Figure1

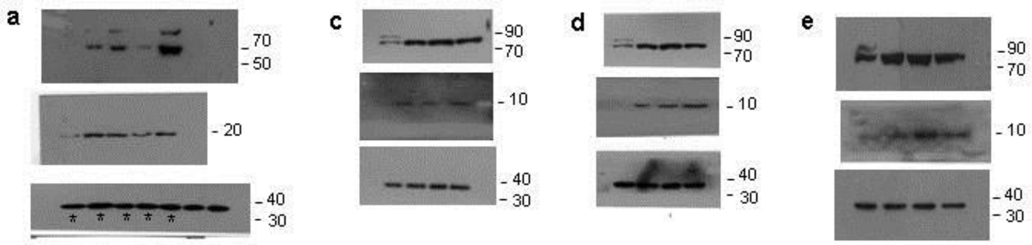

Figure3

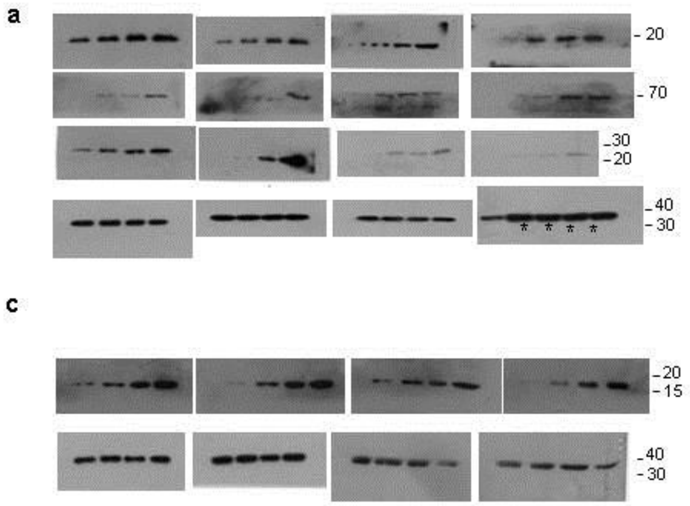

Figure4

c

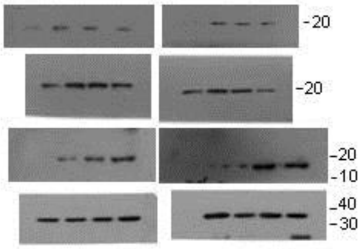

Figure5

c

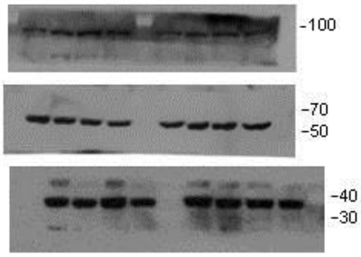

S1

a

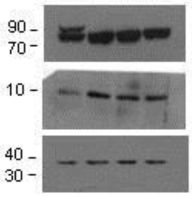

b

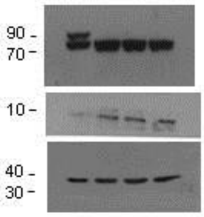

S3

a

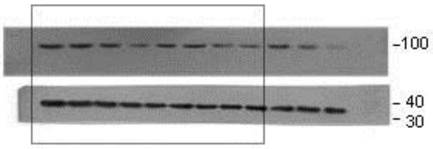

Supplement: Supplementary file 1 — Supplementary information [file 41598_2017_6098_MOESM1_ESM.pdf]
